# Methylation of the NT5E Gene Is Associated with Poor Prognostic Factors in Breast Cancer

**DOI:** 10.3390/diagnostics10110939

**Published:** 2020-11-12

**Authors:** Young Ju Jeong, Hoon Kyu Oh, Hye Ryeon Choi, Sung Hwan Park

**Affiliations:** 1 Department of Surgery, Catholic University of Daegu School of Medicine, Daegu 42471, Korea; shwpark@cu.ac.kr; 2 Department of Pathology, Catholic University of Daegu School of Medicine, Daegu 42471, Korea; ap510@cu.ac.kr; 3 Department of Thyroid and Endocrine Surgery, Thyroid Cancer Center, Severance Hospital, Yonsei University College of Medicine, Seoul 03722, Korea; 2bethere@naver.com

**Keywords:** CD73, *NT5E*, methylation, breast, cancer

## Abstract

Cluster of differentiation (CD) 73, which is encoded by the *NT5E* gene, regulates production of immunosuppressive adenosine and is an emerging checkpoint in cancer immunotherapy. Despite the significance of CD73 in immuno-oncology, the roles of the *NT5E* gene methylation in breast cancer have not been well-defined yet. Therefore, we aimed to investigate the prognostic significance of the *NT5E* gene methylation in breast cancer. The DNA methylation status of the *NT5E* gene was analyzed using pyrosequencing in breast cancer tissues. In addition, the levels of inflammatory markers and lymphocyte infiltration were evaluated. The mean methylation level of the *NT5E* gene was significantly higher in breast cancer than in normal breast tissues. In the analysis of relevance with clinicopathologic characteristics, the mean methylation levels of the *NT5E* gene were significantly higher in patients with large tumor size, high histologic grade, negative estrogen receptor expression, negative Bcl-2 expression, and premenopausal women. There was no difference in disease-free survival according to the methylation status of the *NT5E* gene. We found that the *NT5E* gene methylation was related to breast cancer development and associated with poor prognostic factors in breast cancer. Our results suggest that the *NT5E* gene methylation has potential as an epigenetic biomarker in breast cancer.

## 1. Introduction

Cluster of differentiation (CD) 73 is a glycosylphosphatidylinositol-anchored cell surface protein also known as an ecto-5′-nucleotidase (NT5E), which is encoded by the *NT5E* gene [1]. CD73 has diverse physiological roles including enzymatic and nonenzymatic functions [2]. As a nucleotidase, CD73 plays a critical role in the catabolism of extracellular adenosine monophosphate (AMP) into adenosine, which suppresses antitumor immunity in the tumor microenvironment [3]. In this context, CD73 and downstream adenosine receptors have emerged as novel therapeutic targets in cancer immunotherapy [3,4]. In recent years, the impacts of the CD73-adenosinergic pathway and mechanism of antitumor immunity have increasingly been recognized [5,6,7]. The CD73-derived adenosine contributes to the formation of immunosuppressive tumor microenvironment via activation of immune-checkpoint adenosine A2a receptor on immune cells [5,8]. Targeting the CD73-adenosinergic pathway in preclinical studies showed favorable antitumor effects [8] and early-phase clinical trials showed promising results [7].

The CD73 has been found to be highly expressed in many types of cancer, including breast cancer, colorectal cancer, ovarian cancer, gastric cancer, gallbladder cancer, glioblastoma, melanoma, prostate cancer, ovarian cancer, and non-small-cell lung cancer [9]. Many studies have shown an association between increased expression of CD73 and poor prognosis in cancer patients [5]. However, several studies showed inconsistent results [10], and the prognostic significance of CD73 in breast cancer remains controversial.

The mechanisms that regulate the expression of CD73 in cancer have been elucidated. The expression and function of CD73 is up-regulated in the tumor microenvironment as a result of tissue hypoxia and epithelial–mesenchymal transition [10]. The *NT5E* gene is a hypoxia-inducible factor (HIF) target gene and CD73 transcription is regulated by HIF-1α [8,11]. Also, CD73 expression is regulated by inflammatory mediators including transforming growth factor (TGF)-β, interferons, tumor necrosis factor (TNF), interleukin (IL)-1β, and prostaglandin E2 [8,12,13]. In addition, epigenetic changes can affect the expression of CD73.

Epigenetic modification is an important process that regulates gene expression in normal development and disease including cancer [14,15]. Deoxyribonucleic acid (DNA) methylation is a major epigenetic process, and it is well-established that DNA methylation plays an essential role in carcinogenesis [15]. Recently, with the advent of analytical technology, the methylation status of specific cancer-related genes has been described [16]. In particular, several genes have been shown to be associated with breast cancer and have shown potential diagnostic and prognostic value [17]. However, there have been only few studies on the methylation status of the *NT5E* gene [18,19,20]. A previous study has reported that the *NT5E* gene methylation is associated with favorable outcome in breast cancer [18], but the roles of the *NT5E* gene methylation in breast cancer patients are not defined yet. The purpose of this study was to analyze the DNA methylation status of the *NT5E* gene and to investigate the prognostic significance of *NT5E* gene methylation in breast cancer.

## 2. Materials and Methods

### 2.1. Patients and Materials

Patients who underwent surgery as a primary treatment for breast cancer at Daegu Catholic University Hospital and had frozen surgical specimens were screened. Among them, 47 breast cancer tissues and 10 normal breast tissues were included. The medical records and the pathologic reports of the patients were reviewed, and the clinicopathologic characteristics were evaluated. The written informed consent was obtained from the patients for the use of the patient’s data. The ethics review of the study was waived from the Institutional Review Board at the Daegu Catholic University Hospital according to the deliberation criteria. The approval number is CR-14-096-L and the approval date is 2 July 2014.

Surgical specimens obtained during surgery were managed in two ways. First, immediately after excision of the specimen from the patients, small pieces of tumor tissue and normal breast tissue were collected in sterile collection tubes and frozen at −80 °C. Second, for routine histological examination, samples other than frozen tissue were fixed in formalin and embedded in paraffin (FFPE), and stained with hematoxylin and eosin (H & E). Tumor size, histologic grade of tumor, lymphovascular invasion, regional lymph node metastasis, intratumoral, and peritumor lymphocyte infiltration were evaluated by routine histological examination. Immunohistochemical staining for estrogen receptor (ER), progesterone receptor (PR), human epidermal growth factor receptor 2 (HER2), and Ki-67 were performed on FFPE tissues according to the methods described in our previous study [21].

### 2.2. DNA Extraction and Sodium Bisulfite Treatment

For methylation analysis, DNA was extracted from fresh frozen tissues. Two tissue sections (1-2-mm-thick) were obtained from fresh frozen tissue for each of breast cancer tissue and normal breast tissue. Genomic DNA was extracted and isolated using the QIAamp DNA Mini Kit (Qiagen, Hilden, Germany) following the manufacturer’s instructions. The quality of the purified DNA was verified by gel electrophoresis, and genomic DNA quality control was checked using a NanoDrop spectrophotometer (Thermo Fisher Scientific, Inc., Wilmington, DE, USA). A total of 300 ng of genomic DNA was bisulfite treated with the EZ DNA Methylation-Lightning kit (Zymo Research, Orange, CA, USA), according to the manufacturer’s protocol.

### 2.3. Methylation Analysis

We used pyrosequencing assay for DNA methylation anlaysis, and polymerase chain reaction (PCR) of bisulfite-treated DNA was performed for pyrosequencing analysis. PCR products were amplified using a PCR premix (Enzynomics, Daejeon, Korea) and a PCR instrument (Applied Biosystems, Carlsbad, CA, USA) under the conditions as follows: 95 °C for 10 min, then 45 cycles of 95 °C for 30 s, 54~56 °C for 30 s and 72 °C for 30 s, and then a final extension of 5 min at 72 °C. The annealing temperature was 54 °C for the *NT5E* gene. For PCR of the *NT5E* gene, forward primer was 5′-AGATTAGAAAAGTAAAGAAGGGTAGT-3′ and reverse primer was 5′-CAATTCCTTATTTATTCACAATTAAAACCT-3′. DNA methylation status of the *NT5E* gene was analyzed by using pyrosequencing assay with the Pyromark ID system (Qiagen, Hilden, Germany) and the Pyro-Gold reagent kit (Qiagen, Hilden, Germany) according to the manufacturer’s instructions. Primer for pyroequencing was 5′-GTGTTTTTGTTTTTAGGAG-3′. The methylation status of five CpG sites in the promoter region of the *NT5E* gene in each sample were measured, and the methylation index (MtI) of *NT5E* gene was calculated as the average value of mC/(mC + C) for all of the examined CpGs in target regions.

### 2.4. Analysis of Inflammatory Markers

Tissue microarrays (TMAs) were made with a representative core of each patient’s paraffin block, including breast cancer and normal breast tissue as described in our previous study [21]. Immunohistochemical staining for CD4+ T cells, CD8+ T cells and CD68+ macrophages was performed on TMA sections using the Bond Polymer Intense Detection system (Leica Microsystems, Inc., Buffalo Grove, IL, USA) according to the manufacturer’s protocol with minor modifications. The number of CD4+ and CD8+ T cells and CD68+ macrophages were countered under a microscope and graded as positive if positively stained cells were ≥20 in a TMA section. Intratumoral inflammation was defined as lymphocyte infiltration within the tumor boundary. Peritumoral inflammation was defined as lymphocyte infiltration at the edge of tumor boundary. Intratumoral and peritumoral lymphocyte infiltration was assessed semiquantitatively as follows: 0, no or scant lymphocytes; 1, a few scattered lymphocytic infiltration; 2, scattered lymphocytic aggregation; 3, diffuse and dense aggregation of lymphocytes. Intratumoral and peritumoral inflammation levels 1, 2, and 3 were designated as positive and 0 as negative. 

The levels of inflammatory markers including TNF-α, IL-4, and nuclear factor-kappa B (NF-κB) p50 were analyzed by the levels of ribonucleic acid (RNA) transcript. For reverse transcriptase-PCR (RT-PCR), total RNA was extracted from a small section of fresh frozen tumor tissue using Trizol reagent (#A33250; Invitrogen; Thermo Fisher Scientific, Inc., Wilmington, DE, USA) according to the manufacturer’s protocol. The absorbance of extracted RNA was measured at 260 and 280 nm and quantified. Complementary (c)DNA was generated using a commercial kit (Superscript II RNase H-reverse transcriptase, cat no. 18064071; Invitrogen; Thermo Fisher Scientific, Inc., Wilmington, DE, USA) and RT-PCR was performed as described in our previous study [22]. PCR products were analyzed using agarose gel electrophoresis and ethidium bromide staining.

### 2.5. Statistical Analysis

The methylation status of the *NT5E* gene and the clinicopathologic characteristics were assessed for associations using Fisher’s exact test. The association between inflammatory markers and the methylation status of the *NT5E* gene was also analyzed. Two categorical variables were analyzed using the Chi-square test or Fisher’s exact test, two sample *t*-tests or the non-parametric Mann-Whitney U tests were used for the continuous data. The Kaplan–Meier curve with log-rank test was used to compare the probabilities of overall survival (OS) and disease-free survival (DFS) according to the methylation status of the *NT5E* gene. All statistical analyses were performed using IBM SPSS Statistics ver. 19.0 (IBM, Armonk, NY, USA). All tests were two-sided and a *p*-value of <0.05 was considered to indicate a statistical significance. 

## 3. Results

### 3.1. Methylation Status of the NT5E Gene in Breast Cancer

**Figure 1:**
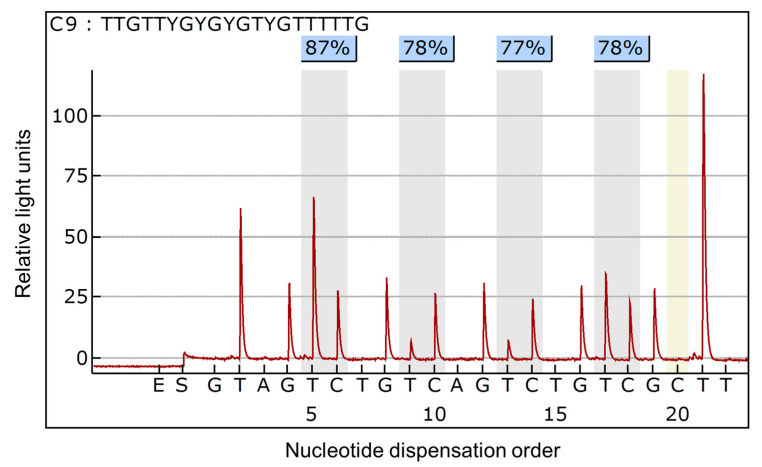
Representative pyrogram of DNA methylation analysis of the *NT5E* gene. The sequence at the top of the pyrogram represents the sequence to be analyzed. Gray shadowing highlights the analyzed CpG sites. The thymine (T) and cytosine (C) peaks indicate unmethylated and methylated C, respectively. Percentage values over the C base are the percent of gene methylation at each site which is defined as the percent of C base. The yellow area indicates the portion of the C added to verify complete conversion of unmethylated C to T.

**Figure 2:**
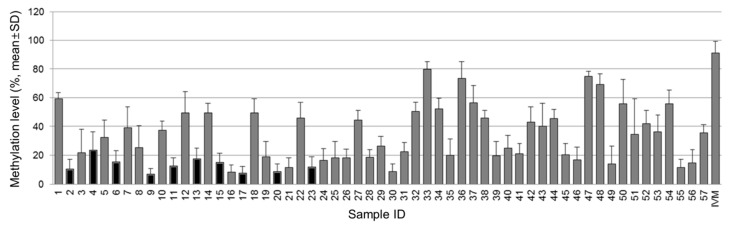
Pyrosequencing data of the *NT5E* gene. Gray box indicates methylation level of the *NT5E* gene in tumor tissue and black box indicates that of normal tissues.

The methylation status of the *NT5E* gene was significantly different between breast cancer tissue and normal breast tissue (*p* < 0.001). The mean methylation level of the *NT5E* gene in tumor tissues was 35.85 ± 18.96% and that in normal breast tissues was 13.11 ± 5.10%. The representative pyrogram of the *NT5E* gene is shown in Figure 1. Figure 2 shows pyrosequencing data of the *NT5E* gene in all tissues, indicating that the mean methylation levels of tumor tissues are relatively higher than those of normal breast tissues.

### 3.2. Association between the Methylation Levels of the NT5E Gene and Inflammatory Markers in Tumor Tissues

CD73 is known to have specific impacts on cancer immunity depending on cell type [3]. We compared the methylation status of the *NT5E* gene according to the expression of inflammatory markers including TNF-α, IL-4, NF-κB p50, CD4+, and CD8+ T cells, CD68+ macrophages, intratumoral and peritumoral inflammation in tumor tissues. There was no significant association between the methylation levels of the *NT5E* gene and analyzed inflammatory markers in breast cancer (Table 1).

### 3.3. Association between the Methylation Levels of the NT5E Gene and the Clinicopathologic Characteristics

Comparison of the methylation status of the *NT5E* gene and clinicopathologic characteristics in tumor tissues showed that the mean methylation frequency of the *NT5E* gene in premenopausal women was significantly higher than in postmenopausal women (*p* = 0.031) (Table 2). In patients with breast cancer larger than 2cm, the level of methylation of the *NT5E* gene was higher than in breast cancer of 2cm or less (*p* = 0.024). The methylation levels were significantly higher in patients with high histologic grade, negative ER expression, and negative B-cell lymphoma 2 (Bcl-2) expression (*p* = 0.01, *p* = 0.01 and *p* = 0.002, respectively). Bcl-2 expression was higher in the luminal A and luminal B subtypes (100% and 87.6%, respectively) compared to the HER2 and basal-like subtypes (33.3% and 66.7%, respectively) (*p* = 0.009). In breast cancer with metastasis, the mean methylation levels of the *NT5E* gene was relatively higher than those without metastasis, but there was no statistical difference (59.53 ± 13.88% and 34.80 ± 18.57%, respectively, *p* = 0.071).

Median follow-up was 93 months (range 24–128 months). There were 6 recurrences and no deaths observed during the follow-up period, except for 8 patients who were lost to follow-up. The 5-year DFS rates were 84.2% (95% CI 77.7–90.7%) for all patients. There was no difference in DFS according to the methylation status of the *NT5E* gene (*p* = 0.146).

## 4. Discussion

Despite many studies on the role of CD73 in the tumor microenvironment, there are only few studies on the methylation of CD73 in cancer [18,19,20]. Herein, we report that the *NT5E* gene methylation is related to breast cancer development and is associated with poor prognostic factors in breast cancer. A single previous study has investigated the *NT5E* gene methylation in breast cancer [18], but to our knowledge, this is the first report of CD73 methylation analysis in fresh frozen human breast cancer tissue and normal human breast tissues. In the current study, the mean methylation level of the *NT5E* gene was significantly higher in breast cancer than in normal breast tissues, which is consistent with previous findings that the *NT5E* CpG island was methylated in specific breast cancer cell lines and was unmethylated in normal breast epithelial cells [18]. Our findings support that aberrant DNA methylation is involved in cancer development by inactivating or repressing gene transcription and affecting chromatin stability [15]. These findings indicate the *NT5E* gene methylation has potential as an epigenetic biomarker in breast cancer.

Adenosine generated by CD73 exerts its biological function via the adenosinergic A2a receptor [7] and suppresses the function of T-lymphocytes [23]. The *NT5E* gene methylation appears to contribute to the downregulation of *NT5E* mRNA expression and subsequent induced anti-tumor immunity in the tumor microenvironment [18,19,20], which also includes some breast cancer cell lines [18]. In this regard, we hypothesized that the *NT5E* gene would be hypomethylated or unmethylated in breast cancer. However, our study showed different results to those expected, suggesting that the *NT5E* gene methylation is associated with tumor cell specificity. It has been previously shown that the *NT5E* gene was frequently methylated in hormone receptor-positive breast cancer, subsets of malignant melanoma, and head and neck squamous cell carcinoma [18,19,20]. In addition, the *NT5E* gene methylation was associated with specific sites of metastasis [18,19,24]. In this study, the mean methylation level of the *NT5E* gene was significantly higher in ER-negative breast cancer and relatively (but not significantly) higher in metastatic breast cancer, although there was no statistical difference. Our results also showed an inverse correlation between the *NT5E* gene methylation and Bcl-2 expression. Bcl-2 has been postulated to be both oncogenic and tumor-suppressive in specific cell types or conditions and reported to be a favorable prognostic factor in breast cancer [25]. Hwang et al. showed that Bcl-2 expression was different according to the molecular subtypes of breast cancer and upregulated in the luminal A and luminal B subtypes compared to the HER2 and basal-like subtypes [25], which is consistent with our results. Taken together, tumor-specific conditions may be specific factors that are associated with the *NT5E* gene methylation.

Epigenetic alterations are emerging as oncologic biomarkers for early cancer detection, treatment selection, monitoring treatment response, and predicting disease outcomes [26,27]. There are several DNA methylation biomarkers in oncology [27]. In breast cancer, *ESR1* methylation can be used as a prognostic and predictive biomarker with diagnostic utility [27]. The *NT5E* DNA methylation also has potential as a biomarker of cancer. Nigro et al. showed that *NT5E* CpG island methylation was associated with low probability of metastasis, non-visceral metastases than visceral metastases, longer DFS, and OS in breast cancer [18]. Wang et al. demonstrated that *NT5E* methylation in malignant melanoma was more common in non-relapsing cases and associated with a lower risk of metastasis to visceral sites and brain [19]. In head and neck squamous cell carcinoma, Vogt et al. reported that the *NT5E* hypomethylation was associated with decreased OS in human papilloma virus (HPV)-positive tumors and increased OS in HPV-negative tumors [20]. Finally, the results of our study, showing the association of the *NT5E* gene methylation with poor prognostic factors such as large tumor size, high histologic grade, negative estrogen receptor expression, and negative Bcl-2 expression in breast cancer, support the utility of the *NT5E* gene methylation as a biomarker of breast cancer. Although our study did not investigate subsequent mechanisms of the *NT5E* gene methylation-related pathway, there are several reasons that may explain our findings that conflict with previous studies. First, Nigro et al. [18] analyzed the *NT5E* gene methylation in three independent breast cancer clinical series with relatively larger sample sizes than ours. Therefore, the selection bias of breast cancer tissue in a small number of breast cancer patients may have influenced the outcome. Second, the recurrence rate of our study was 12.8%, which was lower than that of previous study, 42.1% [18], and there was no death among our patients during the follow-up period. Due to the relatively good prognosis compared to the previous study [18], there may be differences in outcomes for the effect of the *NT5E* methylation on survival. Third, while other genes were also analyzed in previous study [18], we only analyzed the *NT5E* gene methylation. Cross-check of other genes should also be considered to confirm that the effects of the *NT5E* methylation are not the result of a non-specific methylator phenotype [18]. Further clarification requires large-scale, prospective studies with long term follow-up.

Our study has several limitations. First, analysis for independent validation of methylation is lacking. Although we microscopically confirmed tumor tissues and analyzed DNA methylation status using pyrosequencing, tumor microenvironment including tumor infiltrating lymphocytes can influence the methylation status of the *NT5E* gene. Further validation analysis such as methylation-specific PCR is required to confirm the results. Second, we did not analyze the expression of CD73. It is well-recognized that gene expression is regulated epigenetically in cancer [15], and previous studies showed that methylation in the *NT5E* CpG island correlates well with downregulated expression of CD73 in several cancers [18,19,20]. Further studies on the correlation between the *NT5E* gene methylation and CD73 expression is needed to clarify our findings. Third, our results suggest that pathways other than the *NT5E* gene methylation are involved in the regulation of CD73 expression in the tumor microenvironment, but studies on the specific mechanisms involved are lacking. Although we analyzed the association between the methylation status of the *NT5E* gene and inflammatory markers, we did not find any associations. More detailed analyses of the tumor microenvironment are needed to understand the mechanisms involved in the regulation of the *NT5E* gene methylation and CD73 expression in breast cancer. Fourth, our study included a relatively small number of samples. The limited sample size reduces the clinical significance of the study results and limits its clinical application. Further studies of a larger number of breast cancer tissues are needed to provide additional clinically meaningful evidence. Despite these limitations, our results are worthwhile because data on the *NT5E* gene methylation in breast cancer are scarce and our findings provide additional information on the role of CD73 methylation.

In conclusion, we showed that the *NT5E* gene methylation was related to breast cancer development and associated with poor prognostic factors in breast cancer. Our results suggest that methylation of the *NT5E* gene affects carcinogenesis and progression of breast cancer. Further studies are needed to clarify the role of the *NT5E* gene methylation as an epigenetic biomarker and an immunotherapeutic target in breast cancer.

## Figures and Tables

**Table 1:** Association of methylation level of the *NT5E* gene with inflammatory markers in tumor tissues.

Inflammatory Markers		*NT5E* Gene Methylation	
		Mean Levels (%)	*p*-Value
TNF-α	Negative	35.78 ± 21.28	0.996
	Positive	35.84 ± 18.03	
IL-4	Negative	41.63 ± 16.36	0.478
	Positive	36.50 ± 21.36	
NF-κB p50	Negative	27.75 ± 18.08	0.455
	Positive	34.18 ± 17.53	
CD4	Negative	33.35 ± 17.94	0.319
	Positive	39.96 ± 20.17	
CD8	Negative	33.65 ± 15.45	0.765
	Positive	36.17 ± 19.57	
CD68	Negative	33.30 ± 14.84	0.412
	Positive	37.92 ± 21.81	
Intratumoral inflammation	Negative	26.65 ± 16.74	0.115
	Positive	31.16 ± 17.07	
Peritumoral inflammation	Negative	22.62 ± 15.69	0.073
	Positive	37.92 ± 18.99	

TNF, tumor necrosis factor; IL, interleukin; NF-κB, nuclear factor-kappa B; CD, cluster of differentiation.

**Table 2:** Association of methylation level of the *NT5E* gene with clinicopathologic characteristics.

Clinicopathologic Characteristics		*NT5E* Gene Methylation	
		Mean Levels (%)	*p*-Value
Age (years)	<50	39.4 ± 17.88	0.312
	≥50	33.63 ± 19.58	
Menopausal state	Pre-menopausal	43.00 ± 19.73	0.031 *
	Post-menopausal	30.79 ± 16.31	
Stage	I	31.77 ± 3.55	0.4
	II	36.83 ± 21.02	
	III	36.76 ± 21.10	
	IV	59.53 ± 13.89	
Tumor size (cm)	≤2	30.53 ± 16.60	0.024 *
	>2	43.03 ± 19.97	
Nodal involvement	Negative	35.10 ± 17.82	0.735
	Positive	37.06 ± 21.16	
Distant metastasis	Negative	34.80 ± 18.57	0.071
	Positive	59.53 ± 13.88	
Histologic grade	I	23.42 ± 8.15	0.01 *
	II	28.62 ± 15.75	
	III	42.75 ± 19.81	
Lymphovascular Invasion	Negative	36.60 ± 16.06	0.758
	Positive	34.85 ± 22.71	
ER status	Negative	46.09 ± 17.97	0.01 *
	Positive	31.06 ± 17.70	
PR status	Negative	44.25 ± 16.45	0.116
	Positive	33.58 ± 19.16	
HER2 overexpression	Negative	36.18 ± 21.42	0.909
	Positive	36.90 ± 17.17	
Bcl-2	Negative	53.71 ± 19.98	0.002 *
	Positive	32.19 ± 16.76	
Molecular subtype	Luminal A	24.01 ± 13.45	0.099
	Luminal B	38.59 ± 20.56	
	HER2	47.08 ± 18.21	
	Basal-like	36.75 ± 16.29	

ER, estrogen receptor; PR, progesterone receptor; HER2, human epidermal growth factor receptor 2; Bcl-2, B-cell lymphoma 2. * Indicates statistically significant (*p* < 0.05).

## Abbreviations

CD	Cluster of differentiation
NT5E	ecto-5′-nucleotidase
AMP	adenosine monophosphate
HIF	Hypoxia inducible factor
DNA	Deoxyribonucleic acid
TGF	Transformaing growth factor
TNF	Tumor necrosis factor
IL	Interleukin
NF-κB	Nuclear factor-κB
ER	Estrogen receptor
Bcl-2	B-cell lymphoma
DFS	Disease-free survival
OS	Overall survival
HPV	Human papilloma virus
PCR	Polymerase chain reaction
FFPE	Formalin fixed and paraffin embedded
PR	Progesterone receptor
HER2	Human epidermal growth factor receptor 2
TMA	Tissue microarray
RNA	Ribonucleic acid
RT-PCR	Reverse transcriptase-polymerase chain reaction
